# Phytochemical profile and biological activities of the essential oils in the aerial part and root of *Saposhnikovia divaricata*

**DOI:** 10.1038/s41598-023-35656-w

**Published:** 2023-05-29

**Authors:** Bing Li, Zhenmin Yang, Fuying Mao, Qian Wang, Huiyong Fang, Xian Gu, Kaiyan Zheng, Yuguang Zheng, Yunsheng Zhao, Jianming Jiang

**Affiliations:** 1 College of Pharmacy, Hebei University of Chinese Medicine, Shijiazhuang, 050200 China; 2 Traditional Chinese Medicine Processing Technology Innovation Center of Hebei Province, Shijiazhuang, 050200 China; 3 International Joint Research Center on Resource Utilization and Quality Evaluation of Traditional Chinese Medicine of Hebei Province, Shijiazhuang, 050200 China; 4 Experimental Center, Hebei University of Chinese Medicine, Shijiazhuang, 050200 China; 5 Hebei Chemical and Pharmaceutical College, Shijiazhuang, 050026 China

**Keywords:** Plant sciences, Metabolomics

## Abstract

The dried root of *Saposhnikovia divaricata* (Turcz.) Schischk. is popular as a good medicinal material, however the abundant aerial part is often discarded, which caused the waste of resources. In order to exploit resources, the essential oils of the plant aerial part and root were extracted, separately called as VOA and VOR, their chemicals were identified. The tumor necrosis factor-α, interleukin-6, nitric oxide and interleukin-1β were detected to evaluate the oils anti-inflammatory activities. Then, the oils free radical scavenging rates were measured with DPPH, ABTS and hydroxyl free radical. The oils antitumor activities were evaluated with HeLa and HCT-8 cancer cell lines. The results showed the concentrations of VOA and VOR were separately 0.261% and 0.475%. Seventeen components of VOA were identified, accounting for 80.48% of VOA, including phytol, spathulenol, phytone, 4(15),5,10(14)-Germacratrien-1-ol, neophytadiene, etc. Seven components of VOR were determined, representing 90.73% of VOR, consisted of panaxynol, β-bisabolene, etc. VOA and VOR significantly inhibited the secretion of nitric oxide, interleukin-1β, interleukin‐6 and tumor necrosis factor-α, effectively scavenged the DPPH, ABTS and hydroxyl free radicals, and showed significant antiproliferative activity against HeLa and HCT-8. The two oils presented important biological activity, which provided a hopeful utilized basis, and helped to reduce the waste of the aerial non-medicinal resources of *S. divaricata*.

## Introduction

*Saposhnikovia divaricata* (Turcz.) Schischk. (SD) is one of the umbelliferae plants, mainly distributed in China, Japan and Korea. Its dried root is called as Saposhnikoviae Radix, and has been used as a traditional Chinese medicine for more than 2000 years in China^1^.

Saposhnikoviae Radix is now in great demand and mainly comes from the dried root of cultivated SD, but the aerial parts of SD are usually discarded at harvest. This causes a serious waste of resources. Therefore, it is essential to study the chemical composition and pharmacological activity of SD aerial parts^1,2^. It is worth pointing out that in ancient times, in addition to the roots, the leaves, flowers and fruits of SD were also used medicinally. Nowadays, only the root is the medicinal parts. It is worthwhile to study the medicinal properties of its aerial parts to fully utilize SD resources. In order to recycle the discarded aerial resources of SD, we conducted the systematic studies on the compositions and biological activity of the essential oil in the aerial parts of SD (VOA). The essential oils of the plants have been used in naturopathy, alternative medicine, food preservation and pharmaceuticals for thousands of years^3^. The essential oil in Saposhnikoviae Radix (VOR) are relatively complex, and so far, a total of nearly 70 essential oil components have been identified from the roots and fruits of SD using gas chromatography mass spectrometry (GC–MS), including 20 compounds such as panaxynol, α-pinene, β-eudesmol and so on, which have been isolated from the roots^4^. However, VOR was studied mainly on chemical component analysis, its pharmacological activity studies were very few^5,6^.

In order to make effective use of SD resources, it is necessary to conduct scientific studies on VOA and VOR. This study aimed to optimize the extraction method of VOA and VOR, compare their chemical components and biological activities, confirm the similarities and differences between the two essential oils from the roots and aerial parts of SD. The study results provided a promising approach for the appropriate use of the essential oils of SD.

## Results

### Extraction of essential oil

Four different methods were screened to extract SD essential oil (Table 1). Compared with the control group (0.766 g/kg), the group 2 and 3 could significantly increase the oil extraction yield. The yield of 20% KCl extraction was higher than that of steam extraction or 20% NaCl extraction, and reached 0.935 g/kg. The effect of KCl concentration on the extraction yield of VOA was shown in Table 1. With the increase of the KCl concentration, the extraction yield of VOA first increased, and then decreased gradually. The extraction yield without KCl was the lowest, at 0.766 g/kg. When KCl concentration was 5%, the extraction yield of essential oil reached a maximum of 1.283 g/kg. When the concentration of KCl was up to 30%, the extraction yield was only 0.787 g/kg. The analysis of variances indicated that the difference of KCl extractions was significant (*P* < 0.05). Then the different concentrations of KCl were used in the orthogonal experiment of *L*_9_(3^3^), and the maximal extraction yield of essential oil was achieved when material-liquid ratio was 1:15, distillation time was 5 h, and the KCl concentration was at 5%. With the optimized method, the concentrations of VOA and VOR were separately 0.261% and 0.475%.

**Table 1:** The extracted results of the essential oil with different methods.

	Grouping	Group name	extraction yield (g/kg)
Extraction methods	Hydrodistillation extraction	Control	0.766 ± 0.012^c^
	Steam extraction	Group 1	0.457 ± 0.143^d^
	20%NaCl extraction	Group 2	0.907 ± 0.043^b^
	20%KCl extraction	Group 3	0.935 ± 0.087^a^
KCl extraction	5%KCl extraction	Group 3	1.283 ± 0.136^a^
	10%KCl extraction		0.979 ± 0.125^b^
	20%KCl extraction		0.935 ± 0.087^b^
	30%KCl extraction		0.787 ± 0.047^b^

Based on one-way analysis of variance. Values represent means ± standard deviation. The letters of a, b, c and d showed the significant difference for the extraction yields of the essential oil.

### GC/MS analysis of the essential oil

The chemical compositions of VOA and VOR were analyzed using GC–MS techniques. Tables 2 and 3 showed the elution sequence of the discovered compounds on Agilent HP-5MS flexible quartz capillary column, together with their retention times (RT), percentages, and compounds. Twenty-four components from VOA and VOR were identified using GC–MS data and NIST17 data processor. As shown in Table 2, 17 components were identified in VOA, accounting for 80.48% of the total components, composed mainly of sesquiterpenes (32.13%) and diterpenes (31.66%), followed by aliphatic compounds (16.68%). The main constituent was phytol, corresponding 24.56% of VOA (Table 2). Furthermore, VOA displayed moderate levels of spathulenol (9.46%), phytone (8.84%), 4(15),5,10(14)-Germacratrien-1-ol (7.62%), neophytadiene (7.11%). Besides above components, caryophyllene oxide (3.73%), heneicosane (3.4%), Linolenic acid, methyl ester (3.25%), and linoleic acid methyl ester (2.84) exceeded 2% concentration of the total composition of VOA, while the remainder compounds (29.19%) showed low amounts, each one stayed below 2% of the VOA concentration (Table 2).

**Table 2:** Chemical compositions of essential oil from the aerial part of *S. divaricata.*

No.	Compounds^a^	LRI_-Cal_^b^	LRI_-Lit_^c^	ID^d^	References	Relative percentage (%)
1	spathulenol	1575	1576	MS, RI	^7^	9.46 ± 0.37
2	Caryophyllene oxide	1586	1581	MS, RI	^7,8^	3.73 ± 0.49
3	(-)-Spathulenol	1609	1593	MS, RI	^8,9^	1.02 ± 0.45
4	4(15),5,10(14)-Germacratrien-1-ol	1691	1688	MS, RI	^10^	7.62 ± 0.41
5	(2Z,6E)-Farnesol	1699	1696	MS, RI	^11^	1.46 ± 0.63
6	Neophytadiene	1809	1817	MS, RI	^12^	7.11 ± 0.37
7	Phytone	1810	1842	MS, RI	^13^	8.84 ± 0.57
8	Ethyl pentadecanoate	1905	1895	MS, RI	^14^	1.21 ± 0.45
9	Hexadecanoic acid, ethyl ester	1998	1995	MS, RI	^15^	1.12 ± 0.21
10	Panaxynol (Falcarinol)	2009	2034	MS, RI	^7,8^	0.85 ± 0.36
11	Phytol	2103	2114	MS, RI	^10^	24.56 ± 0.42
12	9,12,15-Octadecatrienoic acid, methyl ester, (Z, Z, Z)-	2110	2086	MS, RI	^15^	1.31 ± 0.32
13	Linoleic acid methyl ester	2113	2098	MS, RI	^16^	2.84 ± 0.27
14	Linolenic acid methyl ester	2114	2110	MS, RI	^16^	3.25 ± 0.34
15	Tetracosane	2383	2401	MS, RI	^9^	0.94 ± 0.53
16	Heneicosane	2109	2103	MS, RI	^9^	3.4 ± 0.52
17	(Z)-9-Tricosene	2261	2271	MS, RI	^16^	1.76 ± 0.29
Share of total essential oil (%)		–	–			80.48 ± 4.84

^a^Compounds were listed in order of their retention time on a HP-5MS column.

^b^LRI_-Cal_ = retention indices calculated with n-alkanes (C7-C30) as reference materials.

^c^LRI_-Lit_ = retention indices reported with NIST and/or Adams, Wiley libraries on a HP-5MS column.

^d^ID = Identification methods: MS, by comparison of the mass spectrum with those of the computer mass libraries NIST 17; RI, by comparison of calculated LRI with those reported in the literature.

^e^Tentative identification.

**Table 3:** Chemical composition of the essential oil from the roots of *S. divaricata*.

No	Compounds^a^	LRI_-Cal_^b^	LRI_-Lit_^c^	ID^d^	References	Relative percentage (%)
1	3,3-dimethyl-3H-indazole	1490	–	MS	–	4.48 ± 0.35
2	β-Bisabolene	1496	1508	MS, RI	^7,17^	5.66 ± 0.44
3	Caryophyllenyl alcohol	1569	1569	MS, RI	^18^	2 ± 0.23
4	Viridiflorol	1661	1592	MS, RI	^18^	3.3 ± 0.23
5	Panaxjapyne A^e^	1975	–	MS	^17^	0.81 ± 0.66
6	Panaxynol (Falcarinol)	2009	2034	MS, RI	^7,8^	72.86 ± 0.53
7	Linoleic acid ethyl ester	2153	2182	MS, RI	^16^	1.62 ± 0.59
Share of total essential oil (%)		–	–			90.73 ± 0.72

^a^Compounds were listed in order of their retention time on a HP-5MS column.

^b^LRI_-Cal_ = retention indices calculated with n-alkanes (C7-C30) as reference materials.

^c^LRI_-Lit_ = retention indices reported with NIST and/or Adams, Wiley libraries on a HP-5MS column.

^d^ID = Identification methods: MS, by comparison of the mass spectrum with those of the computer mass libraries NIST 17; RI, by comparison of calculated LRI with those reported in the literature.

^e^Tentative identification.

In VOR, seven compounds were identified, accounting for 90.73% of the total oil. As shown in Table 3, the VOR mainly consisted of Panaxynol, accounting for 72.86% of VOR concentration. Moreover, the VOR appeared with a moderate level of β-Bisabolene (5.66%), followed by 3,3-dimethyl-3H-indazole (4.48%), Viridiflorol (3.3%), Caryophyllenyl alcohol (2%). The other compounds (11.7%) represented lower dosages than 2% of VOR composition (Table 3).

The types and concentrations of VOA components were obviously different from those of VOR. Interestingly, only panaxynol was the common component in VOA and VOR, but its concentration was separately 0.85% and 72.86%.

### Anti-inflammation property

#### Cell viability assay

**Figure 1:**
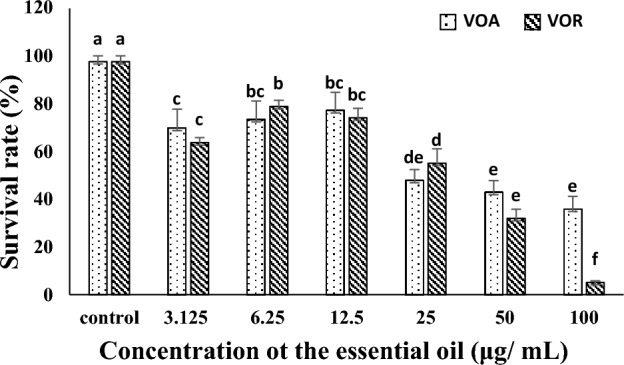
The effect of the essential oil on the survival rate of RAW264.7 cell line. VOA: the essential oil of the aerial part of *S. divaricata*; VOR: the essential oil of the root of *S. divaricata*. The different letters of a-f indicated statistically significant differences (*p* < 0.05).

To determine the cytotoxicity of *S. divaricata* essential oils, RAW 264.7 macrophages were treated with VOA and VOR at various concentrations ranging from 3.125 to 100 μg/mL for 72 h. The changes in cell viability were presented in Fig. 1. The results showed VOA and VOR reduced the cell viability significantly, compared with the control (Fig. 1). The IC_50_ of VOA was calculated to be 35.38 µg/mL for RAW 264.7. VOR showed the IC_50_ of 19.29 µg/mL at the proposed conditions, and remained cell viability below 50% at a concentration of 50–100 µg/mL. 12.5 µg/mL of VOA restored macrophages viability at 77.17 ± 6.23%, and 6.25 µg/mL of VOR held the cell viability at 78.88 ± 2.09%. As shown in Fig. 1, the concentration ranging from 3.125 to 12.5 μg/mL maintained a high survival rate (> 69.76%) for VOA, did not produce significantly different cytotoxicity, and VOR presented the similar effect. Therefore, the low-toxic range was deemed to be from 3.125 µg/mL to 12.5 µg/mL for VOA or VOR, which was selected following the anti-inflammatory assay of RAW 264.7 macrophages.

#### Effects of VOA and VOR on the secretion of NO, IL-6, TNF-α and IL-1β

**Figure 2:**
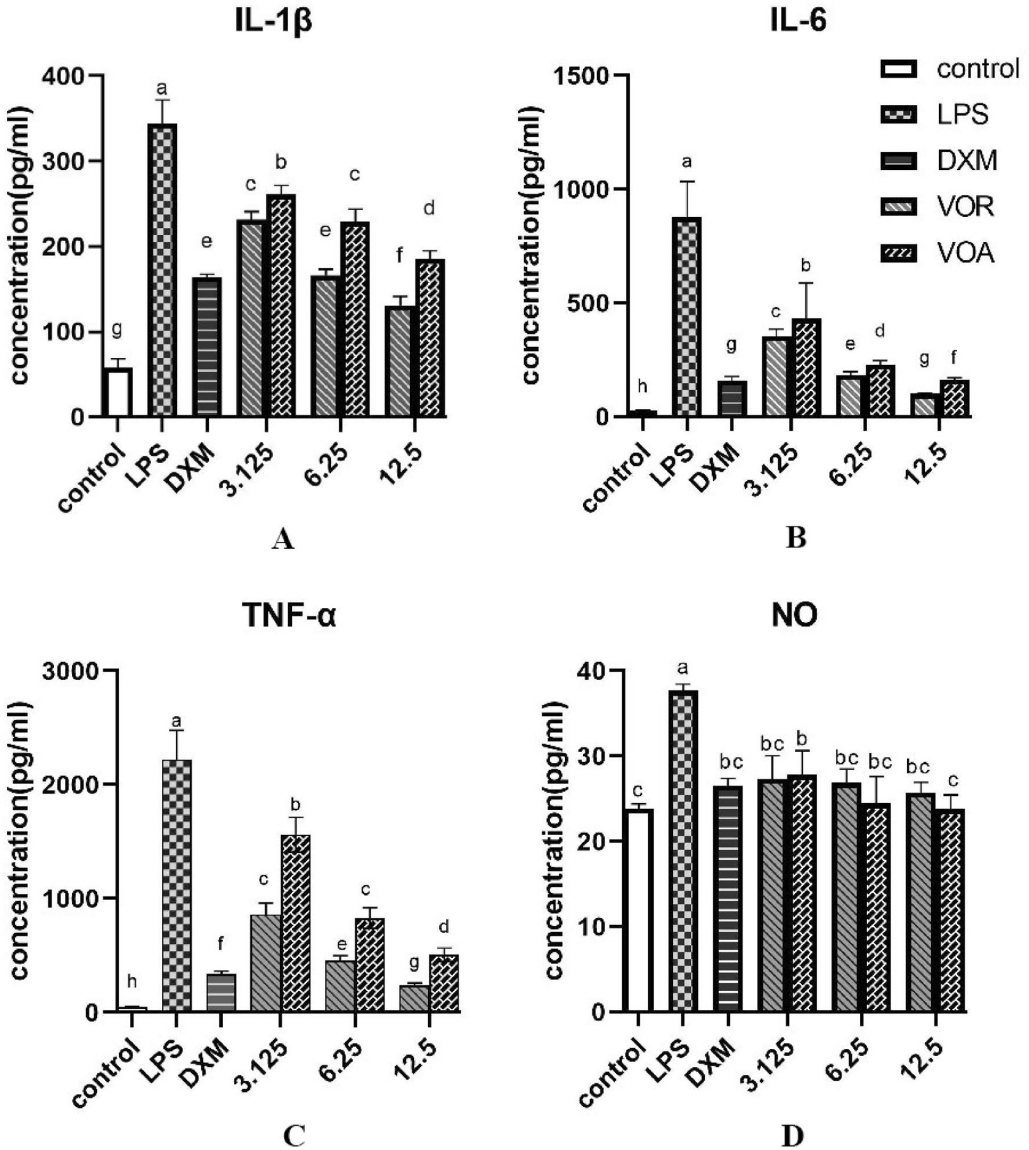
Inhibitory effect of VOA and VOR on cytokines in LPS-stimulated RAW264.7 cells. LPS: lipopolysaccharide; DXM: dexamethasone; VOA: the essential oil of the aerial part of *S. divaricata*; VOR: the essential oil of the root of *S. divaricata*. The different letters of a-h indicated statistically significant differences (*p* < 0.05).

VOA and VOR were assayed for their capacity to decrease NO, IL-6, TNF-α and IL-1β secretion with RAW264.7 macrophages restimulated by 1 µg/mL LPS. As shown in Fig. 2, these inflammatory mediators were substantially increased by LPS but significantly inhibited by VOA and VOR in a dose-dependent manner.

VOA and VOR significantly regulated the production of IL-1β in the macrophages (Fig. 2A) after 24 h of exposure (p < 0.05). The effectiveness of VOA to regulate the production of IL-1β was such that there was an observed reduction of 29.09% at 3.125 µg/mL. At 12.5 µg/mL of VOA, IL-1β was inhibited by 55.35%. IL-1β production was reduced by 39.36% at 3.125 µg/mL of VOR. The concentrations of 6.25 µg/mL and 12.5 µg/mL VOR separately reduced IL-1β production by 62.18% and 74.5%, which was more than the effect of the same concentration of VOA, thus VOR was a more effective IL-1β inhibitor than VOA. However, there was no significant difference for IL-1β between 3.125 µg/mL VOR group and 6.25 µg/mL VOA group, therefore the two groups have the similar inhibitory effect. With the increase of VOR concentration, the IL-1β inhibitory ability of 12.5 μg/mL VOR surpassed that of the 50 µg/mL DXM (*P* < 0.001).

As shown in Fig. 2B, compared with the LPS group, VOR and VOA significantly decreased the secretion of IL-6 in LPS-stimulated RAW264.7 macrophages at 3 different doses. VOA reduced IL-6 secretion by 52.41% at 3.125 μg/ml and 83.99% at 12.5 μg/ml. VOR inhibited the secretion of IL-6 from 61.67% to 91.51%. Interestingly, pretreatment with 12.5 µg/mL VOR significantly reduced IL-6 production, which came up to that of the positive control DXM at 50 μg/ mL. The ability of VOR to inhibit the release of IL‐6 was stronger (*p* < 0.01) than that of VOA at the same concentration. However, 6.25 µg/ml of VOA was stronger inhibitor for IL‐6 than 3.125 μg/ mL of VOR, the ability of 12.5 μg/ml VOA to reduce the secretion of IL‐6 exceeded that of 6.25 μg/mL VOR (*p* < 0.01).

Additionally, Fig. 2C showed the two essential oils effectively inhibited the secretion of TNF-α in RAW264.7 cells induced by LPS at the three doses. VOA inhibited TNF-α production up to 78.79%, whereas VOR inhibited TNF-α production up to 91.38%. There was significant difference (*p* < 0.05) in inhibitory activity of VOA and VOR at the same concentration, and the ability of VOR to inhibit the release of TNF-α was significantly stronger than that of VOA. In particular, at 12.5 µg/mL of VOR, 234.57 pg/ml TNF-α was produced, which was significantly lower (*p* < 0.01) than those of other treated groups, including the positive control DXM group, so 12.5 µg/mL VOR group was the strongest inhibitor of TNF-α. As similar to IL-1β, the TNF-α inhibitory effect of 6.25 µg/ml VOA was close to that of 3.125 μg/ mL VOR, thus, it is possible to use VOA in place of VOR to inhibit the cytokines of IL-1β and TNF-α.

NO has an important effect on inflammation and immune response. Inhibition of excess NO secretion has been used as a test to select anti-inflammatory and immunosuppressive drugs. As shown in Fig. 2D, VOA and VOR significantly reduced the level of NO. The addition of 3.125 µg/mL VOR or VOA to the cells caused a reduction in LPS-induced NO production by 74.87% or 71.73%. The excess secretion of NO was almost totally inhibited at 12.5 μg/ml of VOA, however the inhibitions of VOA and VOR were not significant difference for the different doses, and there was no statistical variation between the DXM and essential oil groups.

### Antioxidant property

**Figure 3:**
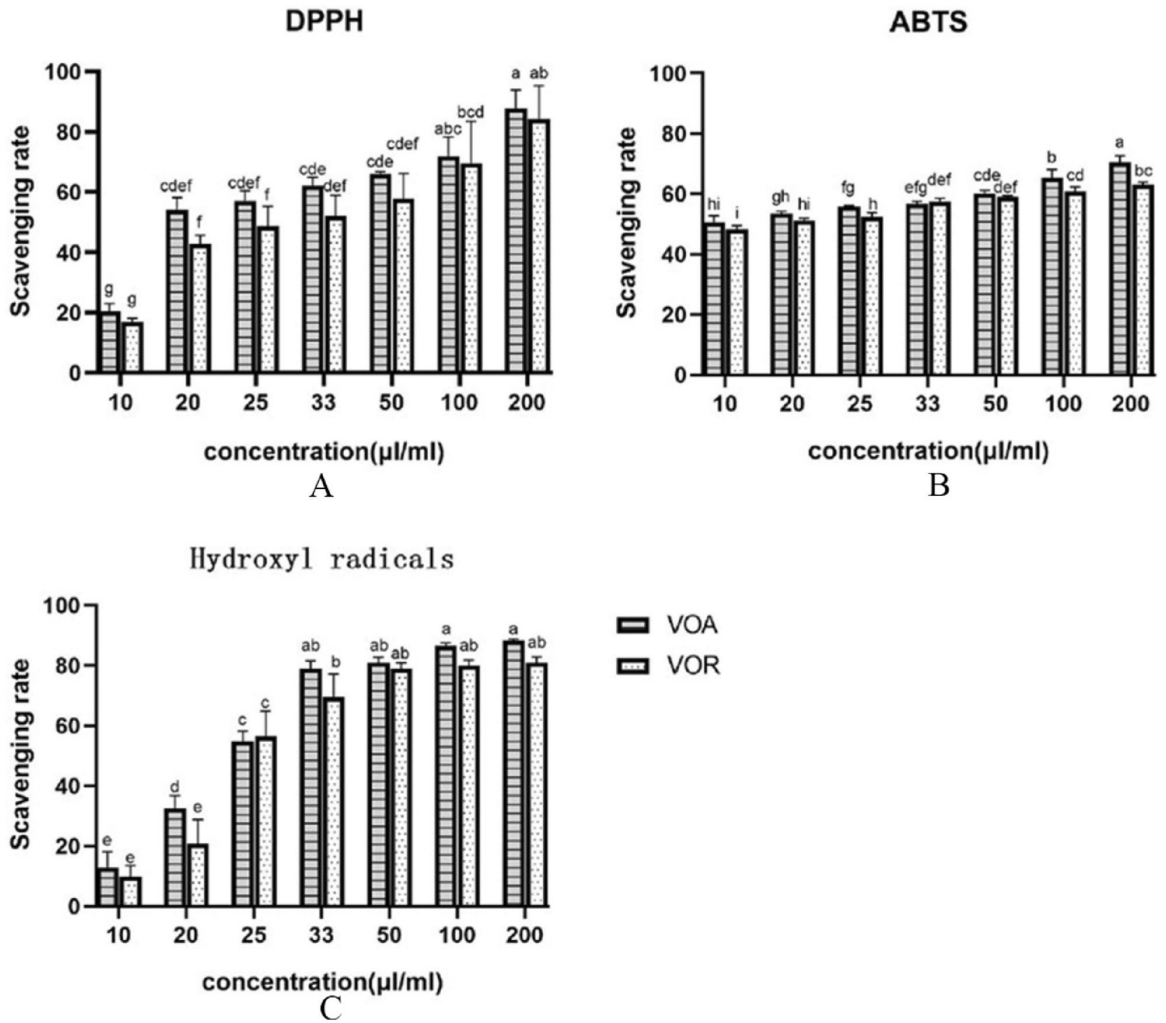
Antioxidant capacity of VOA and VOR. VOA: the essential oil of the aerial part of *S. divaricata*; VOR: the essential oil of the root of *S. divaricata.* The different letters of a-i indicated statistically significant differences (*p* < 0.05).

The free radical scavenging potential of VOA and VOR was investigated for the ABTS, DPPH and Hydroxyl radical. The results were showed in Fig. 3 and Table 4. As shown in Fig. 3A, VOA and VOR separately showed DPPH radical scavenging rates of 20.43%-87.82% and 16.9%-84.35% when their concentrations ranged from 10 to 200 µl/ml, and exhibited good dose-dependent activity. VOA increased the antioxidant capacity compared to VOR at the same concentration, however, the DPPH free radical scavenging activity of the two essential oils was not obviously different from each other at the identical concentration (*p* > 0.05). VOA exhibited higher IC_50_ (23.43 μl/ml) than that of VOR (IC_50_ = 36.58 μl/ml). Lower IC_50_ (μl/ml) indicated the higher antioxidant activity, thus VOA was more potent than VOR for the DPPH free radical scavenging activity.

**Table 4:** Antioxidant IC_50_ values of *S. divaricata* essential oils.

*S. divaricata* essential oil	DPPH	ABTS	Hydroxyl radical
VOA (µl/ml)	23.43 ± 1.71	10.87 ± 1.70	24.42 ± 1.98
VOR (µl/ml)	36.58 ± 9.71	12.50 ± 1.23	29.87 ± 4.86

The potential of VOA and VOR to scavenge ABTS free radicals was assessed, the results were shown in Fig. 3B. VOA and VOR separately showed ABTS radical scavenging rates of 50.78–70.58% and 48.36–63.02% in the concentrations of 10–200 μl/ml, and exhibited good concentration dependent scavenging activity. VOA showed significantly higher ABTS radical scavenging rates than VOR at the concentration of 25, 100 or 200 μl/ml, however, its scavenging activity was not significantly different from VOR at other concentration (*p* > 0.05,). VOA exhibited higher ABTS radical scavenging IC_50_ (10.87 μl/ml) than that of VOR (IC_50_ = 12.50 μl/ml), thus VOA was more effective than VOR for the ABTS free radical scavenging activity. As with DPPH activity, ABTS activity also reflected the ability of the antioxidant potential of VOA and VOR to supply electrons or hydrogen atoms to the ABTS radicals.

The hydroxyl radical scavenging potential of VOA and VOR was presented in Fig. 3C. VOA and VOR separately showed hydroxyl radical scavenging rates of 12.84–88.28% and 9.99–81.02% in the concentrations of 10–200 μl/ml, and exhibited good dose-dependent activity, especially in the concentrations of 10–33 μl/ml. VOR and VOA exhibited strong antioxidant activity in terms of their ability to scavenge hydroxyl radicals, however, their scavenging activities were not significantly different for the concentrations of 50–200 μl/ml (*p* > 0.05,). VOA and VOR possessed strong reducing power, their IC_50_ to scavenge hydroxyl radicals was 24.42 μl/ml and 29.87 μl/ml separately, thus VOA had stronger ability to scavenge hydroxyl radicals compared to VOR.

### Anti-tumor property

**Figure 4:**
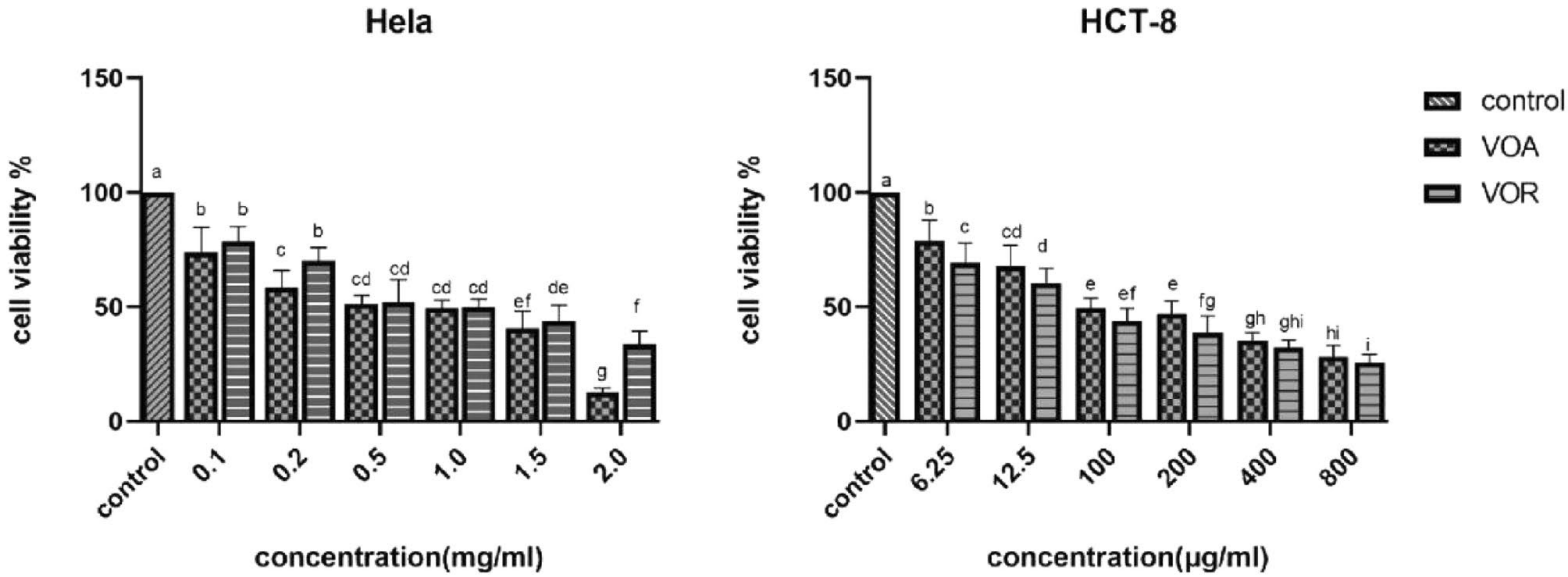
The effect of VOA and VOR on the tumor cells of HeLa and HCT-8. HeLa: cervical cancer cell line; HCT-8: human colon adenocarcinoma cell line; VOA: the essential oil of the aerial part of *S. divaricata*; VOR: the essential oil of the root of *S. divaricata*. The different letters of a-i indicated statistically significant differences (*p* < 0.05).

The cells viabilities of two different cancer cells (HELA and HCT-8) were detected by MTT assay after they were treated with various concentrations VOA or VOR for 24 h. Figure 4 showed the treated groups significantly differed from the control group (*p* < 0.001), and two essential oils displayed significant cytotoxic activity against HeLa and HCT-8 cancer cell lines in a dose-dependent manner. There was a steady decline in the HeLa and HCT-8 cells viabilities as the concentration of the essential oils increased, furthermore, the viabilities of HCT-8 cells showed sharp drop. The antiproliferative IC_50_ values of both tested essential oils were shown in Table 5. The response of the HCT-8 cells to VOA and VOR differed from the HeLa cells. In terms of the cancer cells growth inhibition, it was found that the IC_50_ values of VOA (482.193 µg/mL) and VOR (794.948 µg/mL), applied into HeLa cells, were significantly more than the IC_50_ values of VOA (102.547 µg/mL) and VOR (49.961 µg/mL), subjected into HCT-8 cells, thus, HCT-8 cancer cell lines were more sensitive to the two essential oils than the HeLa cell lines. In contrast to VOR, VOA exhibited the higher antiproliferative effect against HeLa cancer cells with smaller IC_50_ value, and the lower cytotoxic action against HCT-8 with bigger IC_50_ value. While VOR showed the opposite result, and presented potent cytotoxic activity against HCT-8. However, VOA were not significantly different from VOR against HeLa (excluding 0.2 or 2 mg/mL) and HCT-8 (excluding 6.25 or 200 µg/ml) at the same concentration.

**Table 5:** Anti-proliferative IC_50_ values of *S. divaricata* essential oils.

		IC_50_ (µg/mL)	95% confidence limit (µg/mL)	
HeLa	VOR	794.948	688.098	928.613
	VOA	482.193	387.07	593.714
HCT-8	VOR	49.961	40.376	60.966
	VOA	102.547	84.153	125.203

VOA: the essential oil of the aerial part of *S. divaricata*; VOR: the essential oil of the root of *S. divaricata.*

## Discussion

### Extraction and GC/MS analysis of essential oil

Four different methods were elected to measure their extraction yield of essential oil. Steam extraction could avoid the direct contact of water with SD, and the essential oil could be extracted with the penetration of water vapor. Salting can help to reduce the dissolution of some volatile components by water to improve the essential oil yield^19^. KCl-distillation was the best in the four extractions. Its mechanism might be that KCl accelerated the exudation of essential oil from the cell wall of SD, reduced the solubility of essential oil in water. When the concentration of KCl was between 5 and 20%, the dissolvability of the essential oils in water reduced, and more essential oils were steamed out, therefore the extraction yield was significantly increased. However, when KCl was over a certain amount, the addition of KCl could cause the increase of dissolvable substances in the extract, which increased the solution boiling point and reduced the extraction yield of essential oil^20^. Fu et al.^20^ study shows solid–liquid ratio, NaCl concentration and distillation time can optimize the extraction process, which is consistent with our results. However, in our study, the effect of KCl concentration on extraction yield of essential oil was higher than that of NaCl.

Among these compounds in Tables 2 and 3, panaxynol and β-Bisabolene were reported by previous study^5^, the others were not reported. The compositions and proportions of VOA were more complex than those in VOR, which might result in their different biological activities. Similar studies were also reported in other plants, such as *Celtis sinensis*^21^, *Cinnamomum cassia*^22^, *Peucedanum officinale*^23^ and *Peucedanum ruthenicum*^24^. These differences in essential oil compositions of plant different parts probably depended upon the factors of genetic expression and organ function etc.

### Anti-inflammation property

VOA and VOR significantly inhibited the secretion of the studied inflammatory factors. Inflammation is a ubiquitous response to acute or chronic tissue injury^25^. Inflammatory cells can secrete various inflammatory mediators, these mediators take part in the adjustment of the body’s innate immune response, and cause the direct death or apoptosis of target cells, consequently promote the repair of injured tissues. NO, IL-1β, IL‐6 and TNF-α are important indicators of the cellular inflammatory response^26^. Some essential oils, such as those of *Vetiveria zizanioides*, *Thymus vulgaris*, *Artemisia fukudo*, and *Origanum vulgare*, have been reported to inhibit and affect the secretion of NO, IL-1β and IL-6, which was consistent with our study results^27^. VOA and VOR showed significant anti‐inflammatory activity and significantly inhibited the LPS‐stimulated hypersecretion of NO and IL-1β, IL‐6 and TNF-α (Fig. 2). These findings offer good clues for the screening of applicable candidate compounds from VOA and VOR. GC–MS analysis in this study provided details for the chemical composition of VOA and VOR. According to the previous studies, the phytol and panaxynol, separately as the most predominant constituent in VOA and VOR, reduced LPS-induced inflammation by inhibiting TNF-α and IL-1β and IL-6^28–30^. Consistent with the effects observed in mice, the panaxynol extracted from *S. Divaricata* roots can markedly ameliorate acute liver injury caused by LPS and D-galactosamine, which implies it has anti-inflammatory activity in vivo^29^. The anti-inflammatory properties of other main components in VOA and VOR, such as spathulenol, neophytadiene, phytone and β-bisabolene, have also been demonstrated in the previous reports^31–34^. These reported compounds also partly explained the anti-inflammatory effect of VOA and VOR. Therefore, VOA and VOR perhaps could be used as new sources of natural anti-inflammatory drugs in the pharmaceutical industries.

### Antioxidant property

Previous study results showed multiple compounds isolated from the roots of SD possessed significant antioxidant, including polysaccharides, cimifugin, imperatorin, deltoin, and 5-O-methylvisamminol^4^, and the results of the present study increased new antioxidant components (VOA and VOR) for SD. The free radical scavenging capacity of VOA was generally higher than that of VOR. The high free radical scavenging potential of VOA might be due to its main components of olefins and ketones, including phytol, spathulenol, and neophytadiene, which were the effective free radical scavengers^35–38^. VOR had the capacity to suppress the influence of free radicals, might be connected with its certain antioxidant alkynes and olefins such as Panaxynol and β-Bisabolene, which were the main components of VOR, and able to prevent oxidative damage because of their reducing ability^29,39^.

### Anti-tumor property

The previous reports demonstrated that ethanol extracts, coumarins and polysaccharides from SD root and juice of SD had the antiproliferative activity^4,40–42^. The current study confirmed that VOA and VOR also had significant antiproliferative effect on cancer cell lines of HeLa and HCT-8. The cytotoxic activity of VOR might be due to the synergistic effect of its main components, such as Panaxynol, β-disporomycin, etc. Panaxynol is one of the polyacetylene compounds, occur naturally in *S. divaricata, Panax notoginseng, Panax ginseng*, etc., effectively targets both lung cancer stem and non-stem cells in vitro and in vivo, inhibits Hsp90 function by binding to the N-terminal and C-terminal ATP-binding pockets without increasing Hsp70 expression^43^, exerts anti-proliferative effects against human promyelocytic leukemia HL60 cells by induction of apoptosis that was associated with proteolytic cleavage of PKCδ, caspase-3 activation and degradation of PARP^44^. Panaxynol also inhibits the proliferation of HeLa, K562, Wish, Raji, Calu-1, and Vero tumor cells by arresting the cell cycle progression from the G1 transition to the S phase and decreasing the expression of cyclin E mRNA in these tumor cells^45^. β-bisabolene exhibits selective cytotoxic activity for mouse cells (MG1361, Eph4, 4T1) and human breast cancer cells (MDA-MB-231, SKBR3, BT474, MCF-7 and MCF-10A), reduces the growth of transplanted 4T1 mammary tumors in vivo^46^. The concentration of Panaxynol and β-Bisabolene accounted for 78.52% of VOR, so both of them had a great influence on the physiological function of VOR, whose antineoplastic activity also confirmed this in the current study.

The cytotoxic properties of VOA also resulted from the synergistic action of the different constituents, especially the main components including phytol, *spathulenol,* 4(15),5,10(14)-Germacratrien-1-ol, neophytadiene and phytone. Among them, the phytol and *spathulenol* are reported to possess potential antitumor activity. Spathulenol is proved to be cytotoxic against AGS, B16-F10, HepG2, HL-60, K562 and OVCAR-3 cancer cell lines^32,47,48^. Phytol is a potential dietary compound for cancer prevention, in the physiological range (≤ 10 μM), phytol is cytotoxic to various cancer cell lines such as A549, AML12, HeLa, Hs294T, MDA-MB-231, MRC-5, MCF-7, PC-3^49,50^. Phytol can change pathways associated with carcinogenesis, such as enhancing apoptosis and reducing proliferation^50^, however, several evidences showed that phytol at the same dosage had cytotoxicity to breast and brain non-cancer cell lines^50^. The combination between phytol and cytotoxicity/toxicity relies on the dosage/concentration used. Phytol influences the balance of Bcl2 protein, activates the p38 gene, alters Ca^2+^ homeostasis, activates caspases, shifts membrane potential, decreases interleukins, and causes mitochondrial dysfunction, oxidative damage, and epigenetic changes, such as histone deacetylation^41,51^. The 24% of vitro studies showed that phytol induced apoptosis at high dosages, while about 40% of ex vivo researches showed that phytol triggered the generation of reactive oxygen species^51^. The concentration of phytol and spathulenol accounted for 34.02% in VOA, both of them had a great influence on the antiproliferative effects of VOA. The other main compounds in VOA, such as 4(15),5,10(14)-Germacratrien-1-ol, Neophytadiene and phytone, were not reported about their antineoplastic effects, so more researches are needed for finding the potential anticancer agents related to VOA.

## Conclusion

The aerial part and root of *S. divaricata* contained rich essential oil. The chemical compositions and concentrations of the aerial part essential oil were obviously different from those of the root, and the panaxynol was the only common component in the two essential oils. The chemical compositions and proportions of the aerial part essential oil were more complex than those of the root. The two essential oils showed significant anti-inflammatory, antioxidant, and anti-tumor activities in a dose-dependent manner, and their biological activities probably resulted from the synergistic action of the different constituents, especially the main components, including phytol, *spathulenol,* neophytadiene, phytone, panaxynol and β-bisabolene etc. The essential oil of the root was more effective inhibitor for the release of tumor necrosis factor-α, interleukin-1β and interleukin-6 than that of the aerial part, presented more potent cytotoxic activity against HCT-8 cancer cell line. While the essential oil of the aerial part exhibited stronger ability to scavenge DPPH, ABTS, and hydroxyl free radicals, showed the higher antiproliferative effect against HeLa cancer cell line. Both of the essential oils exhibited the potential medicinal value. The current results provided a theoretical basis for the utilization of the essential oils of *S. divaricate*, which would help to exploit the aerial non-medicinal part.

## Methods and materials

### Plant material

The aerial parts (leaves and stems) and roots of SD were collected on 12th October, 2020 in Anguo City, Hebei Province of China, and dried at room temperature. The collection of plant materials was permitted by the owner of the farm and Anguo Agriculture and Rural Bureau. These samples were identified by Prof. Yunsheng Zhao in Hebei University of Chinese Medicine. The voucher specimens of SD were preserved in the herbarium of Hebei University of Chinese Medicine, the serial number was SD-20201012001. The present study complied with relevant institutional, national, and international guidelines and legislation.

### Extraction of essential oil

#### Extraction solvent optimization

The essential oil of SD was extracted with different methods. The hydrodistillation extraction was the control group, and described briefly below: 50 g of SD powder (sieved by No.3) was added to 750 mL of distilled water in a 1000 mL round bottom flask, soaked for 1 h, then a heating mantle was placed under the flask, the flask was connected with an essential oil extractor with the condensation tube. The distilled water was added to fill the scale mark of the extractor. The mixture was heated to boiling, refluxed for 4 h. After the oil–water mixture was clarified in the extractor, the essential oil was collected, and the excess water in it was absorbed by anhydrous Na_2_SO_4_. Three parallel experiments were performed. The extracted essential oil was sealed in amber vials at 4 °C for later use.

In group 1, the process of extraction was modified based on the control group, a distillation flask was placed between the round bottom flask and essential oil extractor, and the SD powder was put in the distillation flask. In group 2, 20% NaCl solution was used instead of the distilled water in the control group. In group 3, 5%, 10%, 20% and 30% KCl solutions were separately replaced the distilled water in the control group. The extraction yields of VOA or VOR were calculated with the following formula:

$${\text{Extraction}}\;{\text{yield}} = {\text{mass}}\;{\text{of}}\;{\text{the}}\;{\text{essential}}\;{\text{oil/mass}}\;{\text{of}}\;{\text{the}}\;{\text{plant}}$$

#### Extraction process optimization

According to the results of the solvent optimization, the orthogonal L_9_ (3^3^) variables were determined to optimize the extraction process. The factors which influenced the essential oil extraction were as follows: the material-liquid ratio, the distillation time and the KCl concentration. They were separately divided into three levels (1, 2 and 3). Factor A was material-liquid ratio, and its three levels were 1:10, 1:15 and 1:20, respectively. Factor B was distillation time (3 h, 4 h and 5 h). Factor C was the KCl concentration (2%, 5% and 8%).

### Analyses and identification of volatile components

After VOA and VOR were separately extracted by optimized parameters in orthogonal design, the analysis of their chemical components was performed by GC–MS procedures using an Agilent 7890B series instrument with an Agilent HP-5MS flexible quartz capillary column (5% phenylmethyl polysiloxane, 0.25 mm × 30 m, film thickness 0.25 μm).

Agilent 7890B series instrument was equipped with detector, autosampler (G4513A series), and NIST17 data processor. The split/splitless injection system used electron ionization mode with electron impact at 70 eV. The carrier gas was helium (99.999% purity), column flow rate 1 ml/min, pressure 151.63 kPa, average flow rate 30 cm/s. To avoid solvent peaks, the mass spectrometer was switched on, the solvent delay time was 3 min, and the shunt ratio was 50:1. The total ion current mode scan range was 50–500 m/z. 230 °C was the injection port temperature, 150 °C was the quadrupole temperature. The programmed ramp-up temperature was as follows: 80 °C was the initial temperature, ramped up to 150 °C at 5 °C/min, then 180 °C at 2 °C/min, then 200 °C at 8 °C/min, then 280 °C at 10 °C/min and maintained for 5 min. The components were identified using the computer Mass Spectra Database (NIST17 database), the retention index (relative to C7–C30 *n*-alkanes, under the same experimental conditions), and literature data. The relative concentration of each component was calculated based on total ion currents. Triple parallel experiments were conducted.

### Cytotoxicity and anti-inflammatory activity

Macrophage RAW264.7 was purchased from the National Collection of Authenticated Cell Culture, and stored at − 80 °C. ELISA kits (TNF-α: SBJ-M0030; IL-6: SBJ-M0657; IL-1β: SBJ-M0027) were purchased from Multi Sciences.

#### Cell culture

Frozen RAW264.7 macrophages were rapidly thawed in a water bath at 37 °C, dissolved with DMEM culture medium, centrifuged, the supernatant was discarded, and then transferred into culture flasks with Dulbecco’s Modified Eagle’s medium (DMEM) (Sigma-Aldrich) supplemented with 10% fetal bovine serum (containing double antibodies), 1% penicillin–streptomycin (100 mg/mL) (Sigma-Aldrich). Cells were incubated at 37℃ in a humidified atmosphere containing 5% CO_2_. The assessment of cell viability was performed by Trypan blue exclusion test (Sigma-Aldrich). After the cells grew to 80% area of the culture flasks bottom by wall attachment, they were digested with trypsin and passaged every 2d at a ratio of 1:2.

#### Cell viability assay

RAW264.7 cells in the logarithmic growth phase were selected, digested with trypsin. The single cell suspension was made with DMEM culture medium containing 10% fetal bovine serum and 1% penicillin–streptomycin, and the cells were seeded in 96-well plates at a density of 2 × 10^5^ cells/well. After the cells were completely attached to the wall, the treated and control groups were set. VOA or VOR was diluted into 3.125, 6.25, 12.5, 25, 50, and 100 μg/mL with DMEM, which were separately added 100 μL into the treated groups cells. The control group cells were added 100 μL DMEM. Each group was composed of 9 wells, after the cells were incubated at 37℃ for 24 h, 10μL CCK8 solution was added to each well to continue incubate for 1 h. After that, the absorbance was measured at 450 nm with VICTOR Nivo Multimode Plate Reader, and the cell survival rate was calculated as follows:

$${\text{Cell}}\;{\text{survival}}\;{\text{rate}}\left( \% \right) = {\text{A}}_{{{\text{treated}}}} /{\text{A}}_{{{\text{control}}}} \times {1}00$$

A_treated_: the absorbance of treated group; A_control_: the absorbance of control group.

All experiments were done in triplicate. The results were expressed by inhibitory concentration IC_50_ value, which constitutes the VOA or VOR concentration required for the occurrence of 50% decrease of cell viability.

#### Anti-inflammatory activity

The anti-inflammatory activity of VOA or VOR was assayed by the measurement of tumor necrosis factor-α (TNF-α), interleukin-6 (IL-6), NO and IL-1β release. RAW264.7 cells were seeded into 96-well plates at a density of 2 × 10^5^ cells/mL and cultured overnight. Then the cells were separately incubated with different concentrations of VOA or VOR (12.5, 6.25, 3.125 μg/mL) and the positive control (Dexamethasone, 50 μg/mL) for 2 h, recorded as A_treated_. These concentrations of VOA, VOR or Dexamethasone were separately prepared with DMEM. After that, the cells were stimulated with lipopolysaccharide (LPS, 1 μg/mL) for another 24 h. Among them, the only LPS-stimulated cells were recorded as A_LPS_, and the cells without VOA, VOR or LPS, were the negative control, recorded as A_control_. The amount of nitrite in the suspension was measured with the Griess reagents, and used as an indicator of NO production^27^. Briefly, 100 µL of the cell supernatant was mixed with 100 µL of Griess reagents and incubated at room temperature for 15 min. Absorbance was determined at 540 nm using an ELISA plate reader. The secretion of IL-6, IL-1β and TNF-α in the supernatants was evaluated using ELISA kits according to the instructions of the manufacturer. The inhibition rate of IL-6, NO, TNF-α or IL-1β was calculated according to the following formula:

$${\text{Inhibition}}\;{\text{rate}}\left( \% \right) = \left[ {{1} - \left( {{\text{A}}_{{{\text{treated}}}} - {\text{A}}_{{{\text{control}}}} } \right)/\left( {{\text{A}}_{{{\text{LPS}}}} - {\text{A}}_{{{\text{control}}}} } \right)} \right] \times {1}00$$

### Antioxidant activity

DPPH Free Radical Scavenging Capacity Assay Kit (A153-1–1), Total antioxidant capacity assay kit (ABTS method, A015-2–1) and Hydroxyl Free Radical assay kit (A018-1–1) were purchased from Nanjing Jiancheng Bioengineering Research Institute.

#### DPPH free radical scavenging assay

VOA or VOR was diluted into 10, 20, 25, 33, 50, 100, 200 μl/ml with methanol, 0.4 mL of each concentration of various dilutions was mixed with 0.6 mL of 0.04% DPPH methanol solution as the sample group. 0.4 mL methanol was mixed with the same DPPH solution as the control group. Each group was incubated for 30 min at 25 ℃ in the dark, the absorbance of the sample or control group was measured at 517 nm and recorded as A_sample_ or A_control_. The clearance of the DPPH was calculated as percent scavenging rate as follows:

$${\text{Scavenging}}\;{\text{rate}}\% = \left( {{\text{A}}_{{{\text{control}}}} - {\text{A}}_{{{\text{sample}}}} } \right)/{\text{A}}_{{{\text{control}}}} \times {1}00$$

The anti-oxidative activity of the specimen was indicated by IC50, which was defined as the concentration that required to reduce the initial concentration of DPPH by 50%. All measured in triplicate.

#### ABTS radical scavenging assay

The ABTS^·+^ cations were produced by mixing 7 mM ABTS stock solution (in methanol) with 2.45 mM K_2_S_2_O_4_ in a volume ratio of 1:1. Then, the mixture was stored in the dark at room temperature for 16 h before use. The mixture was diluted with water to obtain an absorbance of 0.7 ± 0.05 at 734 nm, and called as ABTS solution. After VOA or VOR was diluted into 10, 20, 25, 33, 50, 100, 200 µl/ml with methanol, 10 μL of each concentration of various dilutions was mixed with 190 μL ABTS solution as the sample group. 10 μL methanol was mixed with 190 μL ABTS solution as the control group. Each group was incubated for 6 min at room temperature, the absorbance of sample or control group was measured at 734 nm and recorded as A_sample_ or A_control_, and the percent scavenging rates or IC_50_ calculations were the same with the DPPH free radical scavenging assay.

#### Hydroxyl radical scavenging assay

VOA or VOR was diluted into 10, 20, 25, 33, 50, 100, 200 μl/ml with methanol, 0.2 mL of each concentration of various dilutions was mixed with 0.4 mL FeSO_4_ solution (1.0 mol/L) and 0.2 mL H_2_O_2_ solution (15%), the mixture was shaken well and incubated at room temperature for 10 min, then 2 mL salicylic acid solution was added into, mixed well and left for 30 min as the sample group. Control group comprised of the above solution without essential oil, while in blank group, H_2_O_2_ solution was replaced by methanol. The absorbances of different groups were measured at 550 nm, recorded as A_sample_ and A_control_, and the percent scavenging rates or IC_50_ calculations were the same with the DPPH free radical scavenging assay.

### Antitumor activity

The cervical cancer cell line (HeLa, CL-0101) and human colon adenocarcinoma cell line (HCT-8, CL-0098) were purchased from Procell Life Science & Technology Co., Ltd. They were stored at − 80 °C.

#### Cell culture

The HeLa was cultured in Minimum Essential Medium (MEM, Sigma-Aldrich, St. Louis, MO, USA) supplemented with 10% fetal bovine serum, 1% 100 U/ml penicillin and 100 U/ml streptomycin. The HCT-8 was cultured in Roswell Park Memorial Institute-1640 (RPMI-1640, Sigma-Aldrich, St. Louis, MO, USA) supplemented with 10% fetal bovine serum, 1% 100 U/ml penicillin and 100 U/ml streptomycin, and incubated in a humidified incubator at 37℃ with a 5% CO_2_ atmosphere. The experimental cells were selected in logarithmic growth phase.

#### Cytotoxicity assay

The cell viability was evaluated by using the MTT reduction inhibition assay. The cell density was adjusted to 5 × 10^5^ cells/ml after digestion with 0.25% trypsin. 100 μl cell suspension was seeded in the 96-well flat-bottomed plates and incubated in saturated humidity for 24 h. The essential oil was dissolved in 0.1% dimethyl sulfoxide (DMSO), then diluted into 6.25, 12.5, 100, 200, 400 and 800 µg/mL with RPMI-1640 or 0.1, 0.2, 0.5, 1.0, 1.5 and 2.0 mg/mL with MEM. The HCT-8 cells were treated with 6.25–800 µg/mL of VOA or VOR for 24 h, and the HeLa cells were also treated with 0.1–2.0 mg/ml of VOA or VOR for 24 h, both of them were recorded as treated groups. The control group consisted of treated cells with DMSO, and didn’t contain the essential oil. The cells of treated and control groups were incubated under the same conditions. 20 μl MTT solution (5 mg/mL) was added to each well at an hour before incubation termination, mixed well, and the cells were remained to incubate for 4 h, then the supernatant was discarded. 100 μl DMSO solution was added into each cell well to dissolve the formazan crystals, the cells were protected from light for 10 min, and the absorbance of each well was detected at 570 nm on Multimode Plate Reader. The cell survival rate or IC_50_ calculation was the same with the cell viability assay of macrophage RAW264.7.

### Statistical analysis

All experiments were performed at least three times. Microsoft Excel 2019 was used to calculate the mean concentration and standard deviation of the different data. All data were presented as means ± standard deviation (SD). Analysis of variance (ANOVA) and IC_50_ calculation were carried out using IBM SPSS Statistics 21.0 software (IBM Corp., Armonk, NY, USA). *p* values < 0.05 were considered as statistical significance.

## Data Availability

The datasets presented in the current study are available from the corresponding author on reasonable request.
